# Contextual Factors Associated With County-Level Suicide Rates in the United States, 1999 to 2016

**DOI:** 10.1001/jamanetworkopen.2019.10936

**Published:** 2019-09-06

**Authors:** Danielle L. Steelesmith, Cynthia A. Fontanella, John V. Campo, Jeffrey A. Bridge, Keith L. Warren, Elisabeth D. Root

**Affiliations:** 1 Department of Psychiatry and Behavioral Health, The Ohio State University Wexner Medical Center, Columbus; 2 Rockefeller Neuroscience Institute, Behavioral Medicine and Psychiatry, West Virginia University, Morgantown; 3 Abigail Wexner Research Institute, Nationwide Children’s Hospital, Columbus, Ohio; 4 Departments of Pediatrics, Psychiatry, and Behavioral Health, The Ohio State University, Columbus; 5 College of Social Work, The Ohio State University, Columbus; 6 Department of Geography, The Ohio State University, Columbus

## Abstract

**Importance:**

Understanding geographic and community-level factors associated with suicide can inform targeted suicide prevention efforts.

**Objectives:**

To estimate suicide rates and trajectories, assess associated county-level contextual factors, and explore variation across the rural-urban continuum.

**Design, Setting, and Participants:**

This cross-sectional study included all individuals aged 25 to 64 years who died by suicide from January 1, 1999, to December 31, 2016, in the United States. Spatial analysis was used to map excess risk of suicide, and longitudinal random-effects models using negative binomial regression tested associations of contextual variables with suicide rates as well as interactions among county-level contextual variables. Data analyses were conducted between January 2019 and July 2019.

**Exposure:**

County of residence.

**Main Outcomes and Measures:**

Three-year county suicide rates during an 18-year period stratified by rural-urban location.

**Results:**

Between 1999 and 2016, 453 577 individuals aged 25 to 64 years died by suicide in the United States. Decedents were primarily male (349 082 [77.0%]) with 101 312 (22.3%) aged 25 to 34 years, 120 157 (26.5%) aged 35 to 44 years, 136 377 (30.1%) aged 45 to 54 years, and 95 771 (21.1%) aged 55 to 64 years. Suicide rates were higher and increased more rapidly in rural than in large metropolitan counties. The highest deprivation quartile was associated with higher suicide rates compared with the lowest deprivation quartile, especially in rural areas, although this association declined during the period studied (rural, 1999-2001: incidence rate ratio [IRR], 1.438; 95% CI, 1.319-1.568; *P* < .001; large metropolitan, 1999-2001: 1.208; 95% CI, 1.149-1.270; *P* < .001; rural, 2014-2016: IRR, 1.121; 95% CI, 1.032-1.219; *P* = .01; large metropolitan, 2014-2016: IRR, 0.942; 95% CI, 0.887-1.001; *P* = .06). The presence of more gun shops was associated with an increase in county-level suicide rates in all county types except the most rural (rural: IRR, 1.001; 95% CI, 0.999-1.004; *P* = .40; micropolitan: IRR, 1.005; 95% CI, 1.002-1.007; *P* < .001; small metropolitan: IRR, 1.010; 95% CI, 1.006-1.014; *P* < .001; large metropolitan: IRR, 1.012; 95% CI, 1.006-1.018; *P* < .001). High social capital was associated with lower suicide rates than low social capital (IRR, 0.917; 95% CI, 0.891-0.943; *P* < .001). High social fragmentation, an increasing percentage of the population without health insurance, and an increasing percentage of veterans in a county were associated with higher suicide rates (high social fragmentation: IRR, 1.077; 95% CI, 1.050-1.103; *P* < .001; percentage of population without health insurance: IRR, 1.005; 95% CI, 1.004-1.006; *P* < .001; percentage of veterans: IRR, 1.025; 95% CI, 1.021-1.028; *P* < .001).

**Conclusions and Relevance:**

This study found that suicide rates have increased across the nation and most rapidly in rural counties, which may be more sensitive to the impact of social deprivation than more metropolitan counties. Improving social connectedness, civic opportunities, and health insurance coverage as well as limiting access to lethal means have the potential to reduce suicide rates across the rural-urban continuum.

## Key Points

**Question:**

What are the spatial and temporal trends in suicide rates, how are contextual-level factors associated with suicide, and do these associations vary across the rural-urban continuum?

**Findings:**

This cross-sectional study found that suicide rates in the United States increased from 1999 to 2016, with the greatest increase in rural counties. Deprivation had a disproportionately negative association with suicide rates in rural counties, the presence of gun shops and a higher percentage of uninsured individuals were associated with higher suicide rates, and high social capital was associated with lower suicide rates.

**Meaning:**

Understanding geographical differences in suicide rates and community-level risk and protective factors can inform development and implementation of targeted suicide prevention strategies.

## Teaser

This cross-sectional study estimates suicide rates and trajectories in the United States, assesses associated county-level contextual factors, and explores variation across the rural-urban continuum.

## Introduction

Suicide is a major public health problem in the United States and the tenth leading cause of death, with more than 47 000 individuals dying by suicide in 2017.^1,2^ Despite a national prevention effort initiated in 2015 with the goal of reducing suicide rates 20% by 2025,^3^ suicide rates are trending higher. Analyses from 2018 found that suicide rates increased by more than 30% in 25 states from 1999 to 2016^4^ and nearly 90% of US counties had an increase greater than 20% from 2005 to 2015.^5^ Rural counties consistently have the highest suicide rates and demonstrate the greatest increases over time.^5,6,7,8^

While increasing rates of suicide are well documented, little is known about contextual factors associated with county-level suicide rates. Existing literature documents the association of contextual factors, such as unemployment, poverty, and divorce rates, with suicide rates,^9,10,11^ but it is unclear whether the impact of such factors varies across rural, suburban, and urban communities. Although a few studies suggest that isolation, limited socioeconomic opportunity, and limited access to mental health care in rural communities may contribute to higher suicide rates,^12^ further research is needed to explore the association of contextual factors with county-level suicide rates. Understanding geographical and community-level differences in suicide rates has the potential to inform targeted suicide prevention efforts.

This study examines patterns of suicide in the United States at the county level across the rural-urban continuum during an 18-year period and the association of multiple contextual variables with suicide rates.

## Methods

All individuals aged 25 to 64 years who died from January 1, 1999, to December 31, 2016, and had an underlying *International Statistical Classification of Diseases and Related Health Problems, Tenth Revision *(*ICD*-*10*) cause of death code of suicide (ie, U03*, X60-X84, and Y87.0) were included in this study. We focused on this age range because most studies on mortality trends have focused on this age range.^13,14^ Compressed mortality files obtained from the National Center for Health Statistics National Vital Statistics System with deidentified data were used to identify all suicide decedents and provide information about each decedent’s year of death, sex, age, and county of residence.^15^ Suicides were aggregated by county in 3-year periods to allow for stabilization of suicide rates. Boundary changes over time required some counties be combined (eTable 1 in the Supplement). Population data by county, age, sex, and year for the same age group were obtained through the US Census Bureau website^16^ and summarized across the same 3-year periods.

To examine geographical differences, rural-urban continuum codes (RUCC) were used to classify county types. The RUCC is a 9-category classification system based on county population and adjacency to large metropolitan areas developed by the Economic Research Service of the US Department of Agriculture.^17^ Counties are reclassified after every decennial census, so the 2003 and 2013 RUCCs were used in this study. The 9 categories were collapsed into 4 and classified as follows: (1) large metropolitan counties, (2) small metropolitan counties, (3) micropolitan counties, and (4) rural counties (eTable 2 in the Supplement).

Multiple data sources were used to construct time-varying county-level contextual variables to measure the association of contextual factors with suicide rates during the period studied (eTable 3 in the Supplement). Whenever possible, the years of compressed mortality file data were matched to the years of the other data sources, and missing data were estimated from neighboring year data. Variables included the ratio of primary care physicians to residents, the ratio of psychiatrists to residents, and percentage of veterans from the Area Health Resource File^18^; the ratio of business establishments conducting firearm sales and the ratio of drinking establishments from the Historic Business Database^19^; and the percentage of the population without health insurance from the American Community Survey.^20^ Based on prior literature,^21,22,23,24,25,26,27,28,29^ several indices were also created to measure more complex constructs associated with socioeconomic status and social interaction. Principal component analyses were used to create these indices as described in the eMethods in the Supplement. A deprivation index was modeled after the area deprivation index^30^ and included education, occupation and employment, income, poverty and welfare assistance, and housing tenure and quality subsections. A social fragmentation index included single-person households, percentage of unmarried residents, renter-occupied housing units, and residents who have moved within a year.^21,29^ A social capital index was created from the number of charities, arts and nature facilities, beauty and barber shops, agents and managers, spectator sports, recreation sites, business and political organizations, civic and social associations, and religious organizations.^31^ All 3 index variables were divided into quartiles based on the overall study data, with the first quartile indicating the lowest prevalence of the factor and the fourth quartile, the highest. The first quartile was used as the reference category. Additionally, 3 county-level control variables were included in the analysis: median age, percentage male, and percentage non-Hispanic white. This study followed the Strengthening the Reporting of Observational Studies in Epidemiology (STROBE) reporting guideline for cross-sectional studies.^32^ This study was determined to be exempt from human subjects review by The Ohio State University institutional review board. Informed consent was not required because the study used deidentified data.

### Statistical Analysis

The geographic distribution of suicide was examined for each 3-year period through mapping standardized mortality ratios (SMRs). Standardized mortality ratios were calculated by dividing the number of observed suicides within a county by the expected number of suicides within the same county. Because SMRs can vary drastically based on the size of the population within an area, a Bayesian hierarchical conditional autoregressive model with spatial random effects was used to create spatially smoothed estimates of relative risk in each county for each study period.^33^ The smoothing process accounted for the observed SMR within the county, the national average (global mean) SMR, and neighboring counties’ (local mean) SMRs with varying weighted averages; the smaller the population within a given county, the greater the weight given to the global and local means when calculating the smoothed SMR. Spatial smoothing was done using the diseasemapping package in R version 3.4.3 (The R Foundation). Cartographic displays of smoothed SMRs were created with ArcGIS version 10.3 (Environmental Systems Research Institute).

Longitudinal random-effects models were used to examine the association of county-level suicides with urban or rural residence and other county-level contextual factors. Both the intercept and slope were included as random effects, allowing suicide rates to vary across counties at baseline and during the period studied.^34^ Negative binomial regression with counts of suicides was used to account for overdispersion. The log of the population at risk was also included as an offset variable to allow for interpretation as suicide rates.^35^ We examined 2-way interactions of contextual variables with the 4-category RUCC and with time because we hypothesized that the context of urban, rural, and suburban communities (captured with the RUCC) might affect suicide differently or have diminishing or increasing associations over time. Only interactions that improved model fit were included in the final model. Additional information on the model building process is detailed in the eMethods in the Supplement. Analyses were completed between January 4, 2019, and July 12, 2019. All longitudinal data analyses were done using SAS version 9.4 (SAS Institute). Statistical significance was set at *P* < .05, and all tests were 2-tailed.

## Results

There were a total of 453 577 suicides among US residents aged 25 to 64 years from 1999 to 2016, with the largest proportion occurring in the final 3 years of the study period (90 567 [20.0%]). The majority of decedents were male (349 082 [77.0%]) with 101 312 (22.3%) aged 25 to 34 years, 120 157 (26.5%) aged 35 to 44 years, 136 377 (30.1%) aged 45 to 54 years, and 95 771 (21.1%) aged 55 to 64 years. The median county-level suicide rate increased from 15.0 per 100 000 in 1999 to 2001 to 21.2 per 100 000 in 2014 to 2016 (eTable 4 in the Supplement). Large metropolitan counties accounted for the greatest number of suicides (217 772 [48.0%]), followed by small metropolitan counties (148 716 [32.8%]), micropolitan counties (77 424 [17.1%]), and rural counties (9665 [2.1%]), despite higher suicide rates in rural counties.

### Geographic Distribution of Suicides

**Figure 1.**
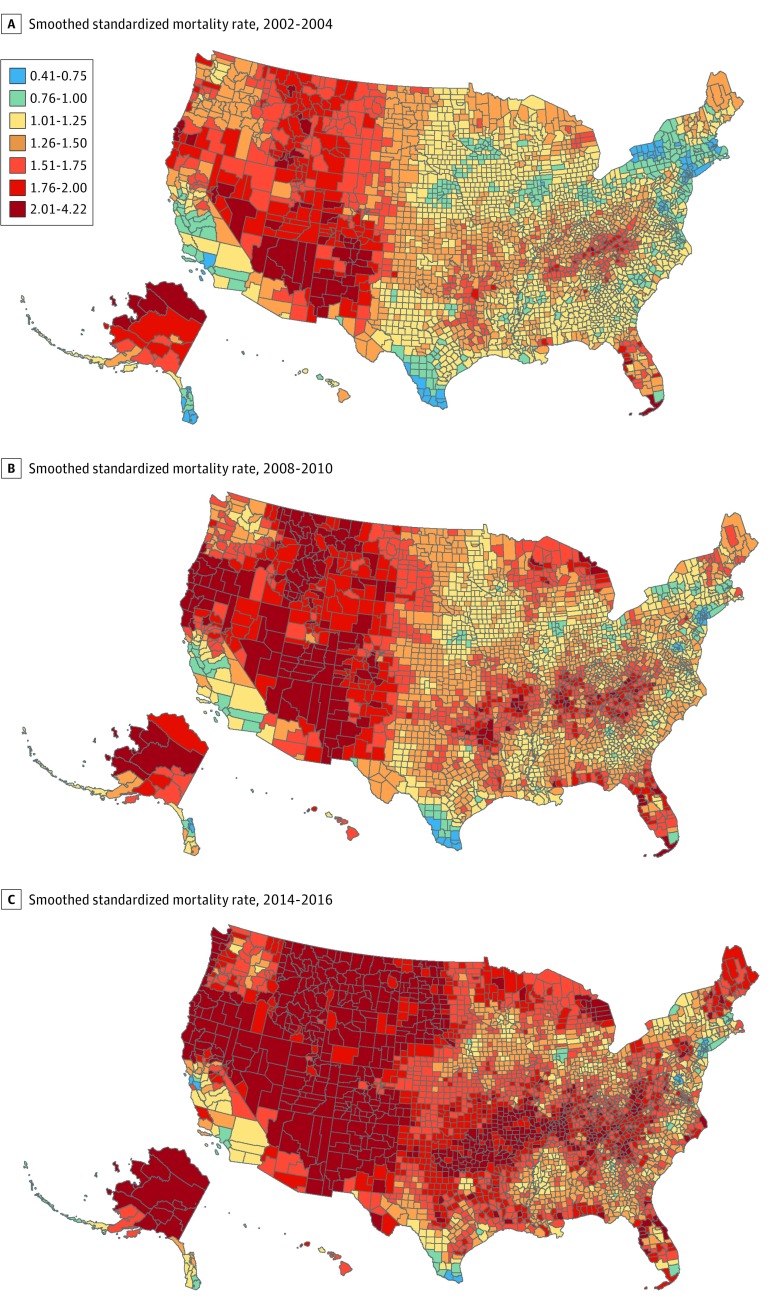
Smoothed Standardized Mortality Ratios in the United States Standardized mortality rates greater than 1.0 correspond to excess risk of suicide, and those less than 1.0 correspond to lower than expected risk of suicide.

Figure 1 shows smoothed SMRs for 3 periods. County-level SMRs ranged from 0.39 to 4.22 during the study, with a mean (SD) of 1.41 (0.37). Ratios of less than 1 correspond to a lower than expected risk of suicide (blue-shaded counties), while ratios over 1 indicate excess risk for suicide (red-shaded counties). As indicated by a greater number of counties in red and dark red in the later time periods, SMRs increased during the period studied. In 1999 to 2001, SMRs ranged from 0.44 to 2.71 with a mean (SD) of 1.18 (0.26) and increased to a range of 0.56 to 4.22 with a mean (SD) of 1.69 (0.40) by 2014 to 2016. Counties with the highest excess risk of suicide tended to be in Western states (eg, Colorado, New Mexico, Utah, and Wyoming), Appalachia (eg, Kentucky, Virginia, and West Virginia), and the Ozarks (eg, Arkansas and Missouri).

### Contextual Factors Associated With Suicide Rates

Summary statistics of county-level contextual variables are described in eTable 5 in the Supplement. More than 40% of counties were classified as micropolitan, followed by approximately 20% as rural, 20% as small metropolitan, and less than 15% as large metropolitan. Veterans represented a median (interquartile range) of up to 13.8% (12.2%-15.4%) of each county’s population, and the median (interquartile range) psychiatrist availability was less than 1 (0-6.0) per 100 000 individuals. Only 2 indices developed for the study showed variation during the study period, with more counties falling into the lowest deprivation quartile and fewer counties falling into the lowest fragmentation quartile over time.

The Table shows the main results of the final longitudinal random-effects model of the association of contextual variables with county-level suicide rates during the study period. Incidence rate ratios (IRRs) and 95% CIs show the association of the independent variables with the suicide rate of a county. Areas with higher levels of community social capital were associated with significantly lower county-level suicide rates, with the highest social capital quartile associated with suicide rate reductions of nearly 10% compared with the lowest quartile (IRR, 0.917; 95% CI, 0.891-0.943; *P* < .001). Counties in the highest social fragmentation quartile were associated with higher suicide rates compared with counties in the lowest quartile (IRR, 1.077; 95% CI, 1.050-1.103; *P* < .001). Similarly, 1-point increases in the percentage of veterans (IRR, 1.025; 95% CI, 1.021-1.028; *P* < .001) and the percentage of individuals without health insurance (IRR, 1.005; 95% CI, 1.004-1.006; *P* < .001) in a county were associated with higher suicide rates.

**Table.** Associations of Contextual Variables With County-Level Suicide Rates From 1999 to 2016

Variables	IRR (95% CI)	*P* Value
Median age^a^	1.004 (1.002-1.006)	<.001
% Non-Hispanic white^a^	1.005 (1.004-1.005)	<.001
% Men^a^	1.004 (0.999-1.008)	.13
Social fragmentation		
Fourth vs first quartile	1.077 (1.050-1.103)	<.001
Third vs first quartile	1.056 (1.035-1.077)	<.001
Second vs first quartile	1.037 (1.020-1.055)	<.001
Social capital		
Fourth vs first quartile	0.917 (0.891-0.943)	<.001
Third vs first quartile	0.936 (0.914-0.958)	<.001
Second vs first quartile	0.961 (0.943-0.979)	<.001
Ratio of psychiatrists to residents, per 100 000 residents	0.999 (0.998-1.000)	.05
Ratio of primary care physicians to residents, per 100 000 residents	1.000 (1.000-1.001)	.10
% Without health insurance	1.005 (1.004-1.006)	<.001
% Veterans	1.025 (1.021-1.028)	<.001
Ratio of drinking establishments to residents, per 100 000 residents	1.000 (1.000-1.000)	.52

Abbreviation: IRR, incidence rate ratios.

^a^ Control variables were global-mean centered; IRRs reflect 1-unit increase from mean.

**Figure 2.**
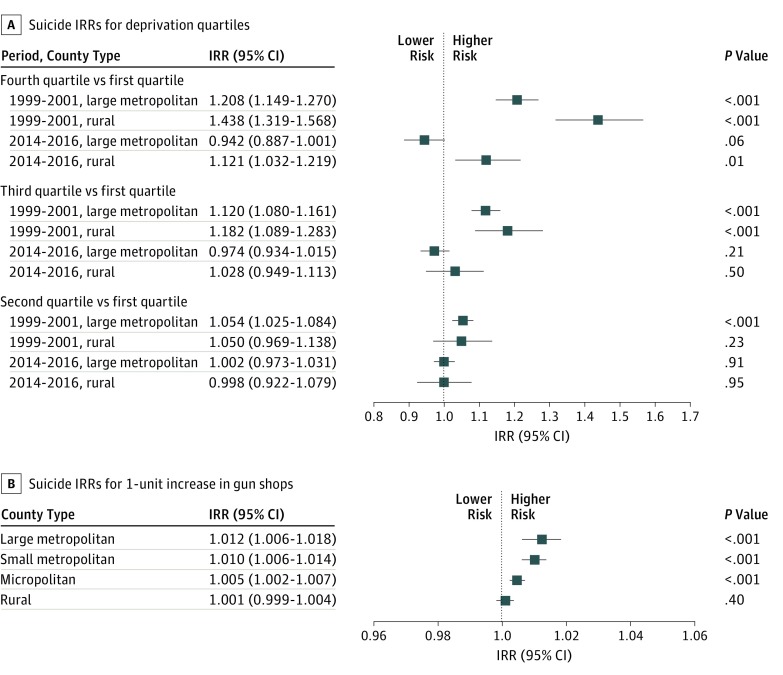
Suicide Incidence Rate Ratios (IRRs) for Deprivation Quartiles and Increases in Gun Shops

To examine whether contextual factors and county-level suicide rates varied across the rural-urban continuum, interactions between contextual variables and RUCCs were examined. Two interactions were significant and retained in the final model, ie, RUCC × deprivation and RUCC × gun shops (Figure 2). Evaluation of the interaction of RUCC and deprivation found that suicide rates in rural counties were disproportionately associated with deprivation compared with large metropolitan counties, especially earlier in the study period and when the highest deprivation quartile was compared with the lowest deprivation quartile (rural, 1999-2001: IRR, 1.438; 95% CI, 1.319-1.568; *P* < .001; large metropolitan, 1999-2001: 1.208; 95% CI, 1.149-1.270; *P* < .001; rural, 2014-2016: IRR, 1.121; 95% CI, 1.032-1.219; *P* = .01; large metropolitan, 2014-2016: IRR, 0.942; 95% CI, 0.887-1.001; *P* = .06) (Figure 2A). Conversely, increases in the presence of gun shops had less association with suicide rates in rural counties than in all other county types (rural: IRR, 1.001; 95% CI, 0.999-1.004; *P* = .40; micropolitan: IRR, 1.005; 95% CI, 1.002-1.007; *P* < .001; small metropolitan: IRR, 1.010; 95% CI, 1.006-1.014; *P* < .001; large metropolitan: IRR, 1.012; 95% CI, 1.006-1.018; *P* < .001) (Figure 2B).

**Figure 3.**
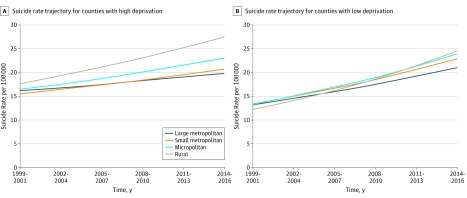
Suicide Rate Trajectories by County Type All continuous variables were set to the county mean, and quartile variables were set to the reference category (first quartile).

Interactions between contextual variables and time were also examined to test temporal variation, and 2 were retained in the final model: RUCC × time and deprivation × time. Rural counties had the most rapid increase in suicide rates compared with metropolitan counties, regardless of deprivation quartile. Figure 3A demonstrates differences in county type when counties in the highest deprivation quartile are considered. The suicide rate in more deprived rural counties was higher than rates in other county types at study outset and increased more rapidly across the study period. Similar findings are illustrated in Figure 3B, where the lowest deprivation quartile counties are considered. While the rural suicide rate in less-deprived counties began lower than rates in other county types, the trajectory was steeper, resulting in higher suicide rates in the lowest deprivation quartile rural counties compared with the lowest deprivation quartile large metropolitan counties in the final period.

**Figure 4.**
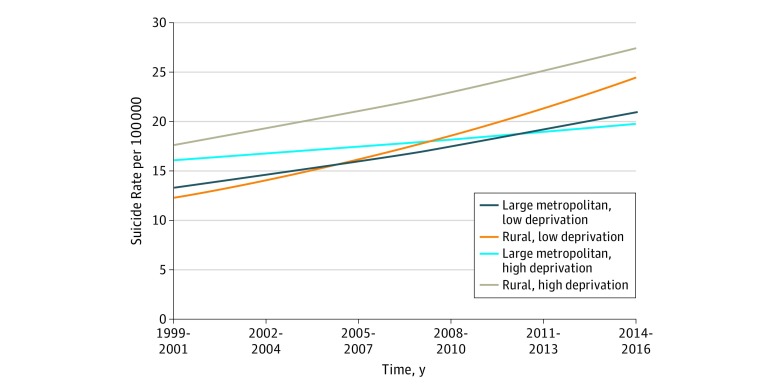
Suicide Rate Trajectories by Deprivation Quartile for Large Metropolitan and Rural County Types All continuous variables were set to the county mean, and quartile variables were set to the reference category (first quartile).

The interaction between deprivation and time shows that the association of deprivation with suicide decreased over time (Figure 4). Counties in the highest deprivation quartile initially had higher suicide rates than other counties, but the trajectory over time was less steep than the lowest deprivation quartile counties. By the final period, there was no real difference in suicide rates across the deprivation quartiles in large metropolitan counties. In rural counties, deprivation had a greater association with suicide rates, so the attenuation of deprivation over time was less noticeable, although the gap between the high and low quartiles narrowed by 2014 to 2016.

### Sensitivity Analysis

Since gun shops were found to be associated with suicides, separate analyses were conducted on firearm suicide deaths and all other methods of suicide. These results can be found in eTable 6 in the Supplement. The gun shop finding reported earlier holds for suicides by firearms, with a slightly larger IRR of 1.033 (95% CI, 1.025-1.042; *P* < .001) in large metropolitan counties compared with 1.003 (95% CI, 1.000-1.006; *P* = .08) in rural counties. However, for suicides by other methods, the presence of gun shops was not statistically associated with suicide except in small metropolitan counties. Even in those counties, the IRR was much smaller (IRR, 1.006; 95% CI, 1.000-1.011; *P* = .04).

## Discussion

This study examined county-level suicide trajectories for adults aged 25 to 64 years in the United States during the 18-year period from 1999 to 2016 across the rural-urban continuum. Our findings confirmed recent reports of increasing suicide rates in the United States^4,5^ and documented a gradient of increasing suicide risk moving from urban to rural settings. The highest observed suicide rates were noted in rural counties, especially those with high deprivation, and suicide rates increased most in rural counties in the western United States, regions of Appalachia, and the Ozarks. These findings are consistent with previous studies demonstrating higher and more rapidly increasing suicide rates in rural areas^6^ and are of considerable interest in light of work by Case and Deaton,^13^ who have documented a persistent annual increase in mortality for white, non-Hispanic men and women in the United States between 1999 and 2013, particularly for those with no more than a high school education.

Our study’s findings of a significant interaction between RUCC and deprivation suggest that individuals living in rural counties may be especially susceptible to the effects of deprivation, such as lower levels of education, employment, and household income. Long-term and persistent poverty appears to be more entrenched and economic opportunities more constrained in rural areas.^36^ Greater social isolation, challenges related to transportation and interpersonal communication, and associated difficulties accessing health and mental health services likely contribute to the disproportionate association of deprivation with suicide in rural counties. National and global trends associated with improvements in the economic outlook of larger cities and towns, such as advances in automation, information technology, and alternative energy, may bypass rural communities, particularly those focused on farming and extractive industries, such as coal mining. Rural counties may lack the flexibility and human capital necessary to adapt to meaningful changes in the broader economy, leading to greater susceptibility to deprivation than more urban or suburban communities.

Although rural counties with high deprivation tend to have the highest overall suicide rates, county-level suicide rates increased less rapidly in counties in the highest deprivation quartile than those in the lowest deprivation quartile. This may be reflective of the conditions in markedly deprived areas, where high levels of deprivation have been persistent for generations. Rural revitalization programs and greater employment opportunities in high-deprivation counties may nevertheless contribute to a reduction in suicide rates and benefit such communities in multiple other ways.

This study’s social fragmentation index included levels of single-person households, unmarried residents, and resident impermanence. Not surprisingly, high social fragmentation was associated with higher suicide rates. Social capital, our variable measuring opportunities for civic engagement, was also associated with suicide rates, with higher social capital availability associated with lower suicide rates. This variable measured the opportunity for people to engage with various organizations and community programs. Consistent with prior research,^37,38^ these findings indicate that greater opportunities for social engagement and connection within a county are associated with lower suicide rates. Programs establishing connectedness and social support within a community are a potential strategy for reducing suicide.^39^ Several programs that include components for building connectedness, such as peer support programs and community engagement activities, have been shown to be effective within bounded settings^40,41,42,43,44^ and could be practically applied to test the effect of enhancing social capital as a means of reducing suicide risk.

This study examined 3 health care variables, but only the health insurance variable was significantly associated with suicide rates, with a larger uninsured population within a county associated with higher suicide rates. This finding is consistent with results of previous studies that associate health insurance coverage with increased mental health treatment^45,46^ and lower suicide rates^47^ and suggests that improving insurance coverage and mental health parity laws may be associated with reduced risks within a community and lower suicide rates.

Consistent with previous studies, a larger percentage of veterans in a county was associated with increased suicide rates, as the rate of suicide among veterans is higher than the general population.^48,49,50,51,52^ This may contribute to the higher rates of suicide in rural areas, where a greater proportion of veterans live.^53^ An estimated 28% of veterans live in rural areas compared with 14% of the general population. In addition, rural communities have had higher rates of recruitment into military service,^54^ which may disrupt family functions and routines and contribute to increased community-wide susceptibility to suicide. This finding highlights the importance of prevention efforts targeting veteran populations and suggests that additional services and supports may be especially relevant for veterans and their families living in rural communities.

The availability of gun shops was also associated with increased suicide rates, highlighting the potential importance of access to lethal means to suicide. Rural households are more likely to own firearms,^55,56^ and some evidence suggests that firearm suicides drive the increased risk of suicide noted in rural areas.^57^ Interestingly, an increase in the number of gun shops appeared to have a greater association with increasing suicide rates in large metropolitan counties than in more rural areas, perhaps suggesting some degree of saturation of access in rural counties relative to more urban areas. This finding was supported by sensitivity analyses of firearm suicide deaths and nonfirearm suicide deaths. Increased accessibility to purchase firearms within a community could be a risk factor for suicide, especially in more urban areas, where gun ownership is less common than in rural areas.^58^ While additional research is necessary, these results provide support for means restriction as a suicide prevention strategy and call attention to projects that engage law enforcement, firearms retailers, and shooting range owners in efforts to prevent suicide.^59,60,61^

### Strengths and Limitations

Strengths of this study include the longitudinal design that incorporates the rural-urban continuum, county-level suicide rates for all 50 states, and the simultaneous use of multiple contextual factors from a variety of sources to understand suicide rates and trajectories. There are limitations as well. First, this is an ecological study that does not allow for interpretation at the individual level. Second, several contextual indices were created, and proxy variables were used to measure general concepts, but these variables may not measure the exact construct of interest (eg, gun shops representing firearm availability). Third, the unit of measure was county. While county is the smallest unit available at the national level, variation within large or densely populated counties may exist for many of the metrics measured. Fourth, national mortality data may misclassify suicide deaths, leading to underestimation. Next, although the sample used in the study was only individuals aged 25 to 64 years, not all contextual variables could be reduced to the same age group. Similarly, because this study only analyzed suicides for ages 25 to 64 years, results are not generalizable to young or elderly adults. Sixth, additional confounding variables that were not controlled for in the analyses may be influencing the results of this study.

## Conclusions

This study examined suicide trajectories during an 18-year period and across the rural-urban continuum. Suicide rates were shown to be increasing most rapidly in rural areas, although all county types saw increases during the period studied. Several contextual factors were associated with suicide rates simultaneously, with social capital being associated with decreased suicide rates. An increase in suicide rates was associated with rural residence, higher deprivation, higher social fragmentation, higher density of gun shops, and a higher percentage of county residents who were veterans and who were uninsured. Study findings suggest that increasing social connectedness, civic opportunities, health insurance coverage, and limiting access to lethal means within communities have the potential to reduce suicide rates across the rural-urban continuum. Suicide rates in rural counties are especially susceptible to deprivation, suggesting that rural counties present special challenges and deserve targeted suicide prevention efforts.

## Notes

Supplement. **eTable 1. **Counties Combined Owing to Boundary Changes **eTable 2. **Rural-Urban Continuum Codes (RUCCs) Description and County Count for 2003 and 2013 **eTable 3. **Description, Year, and Source for Contextual Variables **eTable 4. **Median and Interquartile Range of County-Level Suicide Rates Over Time by Rural-Urban Continuum**** **eTable 5. **Summary of Contextual Variables by Time Period, Reported by County **eTable 6. **Longitudinal Random-Effects Models of Associations of Contextual Variables With Suicide Rates by Method From 1999 to 2016 **eMethods. **Index Creation **eReferences.**
