# Supplementary material for: Contextual Factors Associated With County-Level Suicide Rates in the United States, 1999 to 2016
Source: JAMA Netw Open. 2019 Sep 6;2(9):e1910936. doi: 10.1001/jamanetworkopen.2019.10936 (PMC6735416; doi:10.1001/jamanetworkopen.2019.10936)
Supplement: Supplement. — eTable 1. Counties Combined Owing to Boundary Changes eTable 2. Rural-Urban Continuum Codes (RUCCs) Description and County Count for 2003 and 2013 eTable 3. Description, Year, and Source for Contextual Variables eTable 4. Median and Interquartile Range of County-Level Suicide Rates Over Time by Rural-Urban Continuum eTable 5. Summary of Contextual Variables by Time Period, Reported by County eTable 6. Longitudinal Random-Effects Models of Associations of Contextual Variables With Suicide Rates by Method From 1999 to 2016 eMethods. Index Creation eReferences. [file jamanetwopen-2-e1910936-s001.pdf]

Supplementary Online Content

Steelesmith DL, Fontanella CA, Campo JV, Bridge JA, Warren KL, Root ED. Contextual factors associated with county-level suicide rates in the United States, 1999 to 2016. *JAMA Netw Open*. 2019;2(9):e1910936. doi:10.1001/jamanetworkopen.2019.10936

eTable 1. Counties Combined Owing to Boundary Changes

eTable 2. Rural-Urban Continuum Codes (RUCCs) Description and County Count for 2003 and 2013

eTable 3. Description, Year, and Source for Contextual Variables

eTable 4. Median and Interquartile Range of County-Level Suicide Rates Over Time by Rural-Urban Continuum

eTable 5. Summary of Contextual Variables by Time Period, Reported by County

eTable 6. Longitudinal Random-Effects Models of Associations of Contextual Variables With Suicide Rates by Method From 1999 to 2016

eMethods. Index Creation

eReferences.

This supplementary material has been provided by the authors to give readers additional information about their work.

© 2019 Steelesmith DL et al. *JAMA Network Open.*

eTable 1. Counties Combined Owing to Boundary Changes

State	County FIPS	Year of Boundary Changes
Alaska		
	02130, 02195, 02198, 02201, 02275, and 02280	2008
	02230, 02105, and 02232	2007
Virginia		
	51515 and 51019	2013
	51005 and 51560	2001

© 2019 Steelesmith DL et al. *JAMA Network Open.*

eTable 2. Rural-Urban Continuum Codes (RUCCs) Description and County Count for 2003 and 2013

Collapsed	Code	Description	2003 Number of Counties	2013 Number of Counties
**Metro**				
Large Metropolitan	1	Counties in metro areas of 1 million population or more	413	432
Small Metropolitan	2	Counties in metro areas of 250,000 to 1 million population	325	379
	3	Counties in metro areas of fewer than 250,000 population	351	356
**Non-metro**				
Micropolitan	4	Urban population of 20,000 or more, adjacent to a metro area	218	214
	5	Urban population of 20,000 or more, not adjacent to a metro area	105	92
	6	Urban population of 2,500 to 19,999, adjacent to a metro area	609	593
	7	Urban population of 2,500 to 19,999, not adjacent to a metro area	450	433
Rural	8	Completely rural or less than 2,500 urban population, adjacent to a metro area	235	220
	9	Completely rural or less than 2,500 urban population, not adjacent to a metro area	435	424

© 2019 Steelesmith DL et al. *JAMA Network Open.*

eTable 3. Description, Year, and Source for Contextual Variables

Category	Variable	Year	Source
**Socioeconomic Deprivation**<br>(22 variables combined into single index using principal component analysis)			
Education			
	Population aged 25+ with < 9 years of education, %	2000, 2005-2009, 2007-2011, 2010-2014, 2013-2017	Census, 5-year ACS
	Population aged 25+ with at least a high school diploma, %	2000, 2005-2009, 2007-2011, 2010-2014, 2013-2017	Census, 5-year ACS
Occupation and employment			
	Employed persons aged 16+ in white collar occupations, %	2000, 2005-2009, 2007-2011, 2010-2014, 2013-2017	Census, 5-year ACS
	Civilian labor force population aged 16+ unemployed, %	2000, 2005-2009, 2007-2011, 2010-2014, 2013-2017	Census, 5-year ACS, AHRF
Income			
	Median family income, $	2000, 2005-2009, 2007-2011, 2010-2014, 2013-2017	Census, 5-year ACS, AHRF
	Gini coefficient	2000, 2005-2009, 2007-2011, 2010-2014, 2013-2017	5-year ACS
	Median home value, $	2000, 2005-2009, 2007-2011, 2010-2014, 2013-2017	Census, 5-year ACS
	Median gross rent, $	2000, 2005-2009, 2007-2011, 2010-2014, 2013-2017	Census, 5-year ACS
	Median monthly mortgage, $	2000, 2005-2009, 2007-2011, 2010-2014, 2013-2017	Census, 5-year ACS
	Median annual real estate taxes, $	2000, 2005-2009, 2007-2011, 2010-2014, 2013-2017	Census, 5-year ACS
Poverty and welfare assistance			
	Families below poverty level, %	2000, 2005-2009, 2007-2011, 2010-2014, 2013-2017	Census, 5-year ACS, AHRF
	Population below 150% of poverty threshold, %	2000, 2005-2009, 2007-2011, 2010-2014, 2013-2017	Census, 5-year ACS
	Single-parent households with children aged < 18, %	2000, 2005-2009, 2007-2011, 2010-2014, 2013-2017	Census, 5-year ACS
	Households receiving supplemental security income, %	2000, 2005-2009, 2007-2011, 2010-2014, 2013-2017	Census, 5-year ACS
	Households receiving public assistance income, %	2000, 2005-2009, 2007-2011, 2010-2014, 2013-2017	Census, 5-year ACS
Housing tenure and quality			
	Owner-occupied housing units, %	2000, 2005-2009, 2007-2011, 2010-2014, 2013-2017	Census, 5-year ACS
	Households without a motor vehicle, %	2000, 2005-2009, 2007-2011, 2010-2014, 2013-2017	Census, 5-year ACS

© 2019 Steelesmith DL et al. *JAMA Network Open.*

	Households without a telephone, %	2000, 2005-2009, 2007-2011, 2010-2014, 2013-2017	Census, 5-year ACS
	Occupied housing units without complete plumbing, %	2000, 2005-2009, 2007-2011, 2010-2014, 2013-2017	Census, 5-year ACS
	Households with more than 1 person per room, %	2000, 2005-2009, 2007-2011, 2010-2014, 2013-2017	Census, 5-year ACS
	Median number of rooms per housing unit	2000, 2005-2009, 2007-2011, 2010-2014, 2013-2017	Census, 5-year ACS
	Housing units with 4 or more bedrooms, %	2000, 2005-2009, 2007-2011, 2010-2014, 2013-2017	Census, 5-year ACS
**Social Fragmentation**<br>(4 variables combined into single index using principal component analysis)			
	Residents not in the same house 1 year ago, %	2000, 2005-2009, 2007-2011, 2010-2014, 2013-2017	Census, 5-year ACS
	Single person households, %	2000, 2005-2009, 2007-2011, 2010-2014, 2013-2017	Census, 5-year ACS
	Unmarried residents (includes single, separated, and divorced), %	2000, 2005-2009, 2007-2011, 2010-2014, 2013-2017	Census, 5-year ACS
	Renter-occupied housing units, %	2000, 2005-2009, 2007-2011, 2010-2014, 2013-2017	Census, 5-year ACS
**Social Capital**<br>(9 variables combined into single index using principal component analysis)			
	Charities (foundations and public charities) per 100,000 persons	1999-2015	NCCS
	Arts and nature facilities (libraries, museums, historical sites, zoos and botanical gardens, nature parks) per 100,000 persons	1999-2016	CBP
	Beauty facilities (barbers, beauty salon, nail salon) per 100,000 persons	1999-2016	CBP
	Agents (promotors, agents, managers) per 100,000 persons	1999-2016	CBP
	Spectator sports (racetracks, sports clubs, other spectator sports) per 100,000 persons	1999-2016	CBP
	Recreation sites (public golf courses, physical fitness facilities, bowling centers, skiing, marinas) per 100,000 persons	1999-2016	CBP
	Business and political organizations (business associations, professional, labor, and	1999-2016	CBP

© 2019 Steelesmith DL et al. *JAMA Network Open.*

	political organizations) per 100,000 persons		
	Civic and social associations per 100,000 persons	1999-2016	CBP
	Religious organizations per 100,000 persons	1999-2016	CBP
**Health Providers/Services**			
	Number of psychiatrists per 100,000 persons	1999-2016	AHRF
	Number of primary care physicians/general and family doctors per 100,000 persons	1999-2016	AHRF
	Individuals without health insurance between ages 18 and 64, %	2000, 2005-2016	AHRF
**Firearms**<br>(5 variables summed and averaged per 100,000 individuals)			
	NAICS code 45111001: Ammunition	1999-2016	US Historical Business
	NAICS code 45111002: Ammunition reloading equipment and supplies	1999-2016	US Historical Business
	NAICS code 45111023: Guns and gunsmiths	1999-2016	US Historical Business
	NAICS code 45111024: Gunsights, scopes, and mounts	1999-2016	US Historical Business
	NAICS code 45111076: Black powder guns and supplies	1999-2016	US Historical Business
**Alcohol**<br>(9 variables summed and averaged per 100,000 individuals)			
	NAICS code 72241001: Bars	1999-2016	US Historical Business
	NAICS code 72241002: Cabarets	1999-2016	US Historical Business
	NAICS code 72241003: Cocktail lounges	1999-2016	US Historical Business
	NAICS code 72241004: Comedy Clubs	1999-2016	US Historical Business
	NAICS code 72241005: Discotheques	1999-2016	US Historical Business
	NAICS code 72241006: Nightclubs	1999-2016	US Historical Business
	NAICS code 72241007: Nightclubs Information Service	1999-2016	US Historical Business
	NAICS code 72241008: Pubs	1999-2016	US Historical Business
	NAICS code 72241009: Karaoke Clubs	1999-2016	US Historical Business

© 2019 Steelesmith DL et al. *JAMA Network Open.*

**Veterans**			
	Percent of veterans	1999-2016	AHRF
**Control Variables**			
	Median age	2000, 2005-2009, 2007-2011, 2010-2014, 2013-2017	Census, 5-year ACS
	Percent male	1999-2016	AHRF
	Percent non-Hispanic white	1999-2016	AHRF

Note. ACS = American Community Survey, CBP = County Business Patterns, AHRF = Area Health Resource File, NAICS = North American Industry Classification System, NCCS = National Center of Charitable Statistics

© 2019 Steelesmith DL et al. *JAMA Network Open.*

eTable 4. Median and Interquartile Range of County-Level Suicide Rates Over Time by Rural-Urban Continuum

	1999-2001	2002-2004	2005-2007	2008-2010	2011-2013	2014-2016
**Suicide Rates**						
Overall, per 100,000	14.95 (9.83-20.56)	15.95 (10.96-22.13)	16.47 (11.42-22.83)	17.95 (12.73-24.49)	19.55 (13.87-26.99)	21.21 (14.76-28.48)
Large metropolitan, per 100,000	13.21 (9.97-16.69)	14.12 (10.86-18.31)	14.67 (11.86-18.86)	16.15 (13.02-20.05)	16.97 (13.32-21.53)	17.63 (14.10-22.98)
Small metropolitan, per 100,000	14.88 (11.05-18.36)	15.64 (12.35-20.08)	16.86 (13.21-21.51)	17.89 (13.72-22.43)	19.36 (15.24-24.59)	20.06 (16.26-26.47)
Micropolitan, per 100,000	15.95 (10.85-21.29)	17.04 (12.03-23.27)	17.28 (11.97-23.50)	19.01 (13.62-25.24)	20.94 (14.77-27.94)	22.60 (16.14-29.13)
Rural, per 100,000	14.52 (0.00-25.67)	14.33 (0.00-26.37)	15.41 (0.00-28.12)	17.45 (4.97-31.62)	20.18 (6.94-34.15)	22.05 (7.03-37.00)

© 2019 Steelesmith DL et al. *JAMA Network Open.*

eTable 5. Summary of Contextual Variables by Time Period, Reported by County

	1999-2001 (N=3,137)	2002-2004 (N=3,138)	2005-2007 (N=3,138)	2008-2010 (N=3,138)	2011-2013 (N=3,138)	2014-2016 (N=3,138)
**Contextual Variables**						
Median age, median (IQR)	37.4 (35.2-39.7)	37.4 (35.2-39.7)	39.4 (36.6-42.5)	40.1 (37.3-43.1)	40.7 (37.6-43.7)	41.2 (37.9-44.2)
Percent non-Hispanic white, median (IQR)	89.3 (71.5-96.0)	88.7 (70.4-95.7)	87.6 (69.1-95.1)	86.4 (67.6-94.3)	85.1 (66.2-93.6)	84.2 (65.1-92.9)
Percent male, median (IQR)	49.2 (48.5-50.0)	49.3 (48.7-50.1)	49.4 (48.7-50.2)	49.5 (48.8-50.2)	49.6 (48.9-50.5)	49.6 (49.0-50.5)
RUCC						
Large metropolitan, n (%)	413 (13.2)	414 (13.2)	414 (13.2)	432 (13.8)	432 (13.8)	432 (13.8)
Small metropolitan, n (%)	675 (21.5)	675 (21.5)	675 (21.5)	734 (23.4)	734 (23.4)	734 (23.4)
Micropolitan, n (%)	1380 (44.0)	1380 (44.0)	1380 (44.0)	1332 (42.5)	1332 (42.5)	1332 (42.5)
Rural, n (%)	669(21.3)	669 (21.3)	669 (21.3)	640 (20.4)	640 (20.4)	640 (20.4)
Deprivation quartiles						
1st quartile (low), n (%)	339 (10.8)	340 (10.8)	877 (28.0)	975 (31.1)	985 (31.4)	1194 (38.1)
2nd quartile, n (%)	722 (23.0)	722 (23.0)	833 (26.6)	823 (26.2)	795 (25.3)	812 (25.9)
3rd quartile, n (%)	881 (28.1)	881 (28.1)	728 (23.2)	739 (23.6)	763 (24.3)	710 (22.6)
4th quartile (high), n (%)	1195 (38.1)	1195 (38.1)	700 (22.3)	601 (19.2)	595 (19.0)	422 (13.5)
Social Fragmentation quartiles						
1st quartile (low), n (%)	1488 (47.4)	1488 (47.4)	541 (17.2)	527 (16.8)	354 (11.3)	309 (9.9)
2nd quartile, n (%)	831 (26.5)	831 (26.5)	810 (25.8)	802 (25.6)	734 (23.4)	701 (22.3)
3rd quartile, n (%)	486 (15.5)	487 (15.5)	904 (28.8)	898 (28.6)	962 (30.7)	970 (30.9)
4th quartile (high), n (%)	332 (10.6)	332 (10.6)	883 (28.1)	911 (29.0)	1088 (34.7)	1158 (36.9)
Social Capital quartiles						
1st quartile (low), n (%)	801 (25.5)	787 (25.1)	753 (24.0)	758 (24.2)	815 (26.0)	794 (25.3)
2nd quartile, n (%)	791 (25.2)	764 (25.4)	795 (25.3)	775 (24.7)	791 (25.2)	795 (25.3)
3rd quartile, n (%)	811 (25.9)	813 (25.9)	777 (24.8)	787 (25.1)	762 (24.3)	755 (24.1)
4th quartile (high), n (%)	734 (23.4)	774 (24.7)	813 (25.9)	818 (26.1)	770 (24.5)	794 (25.3)
Psychiatrist ratio per 100,000 residents, median (IQR)	.55 (0-6.04)	.64 (0-6.04)	.75 (0-6.01)	0 (0-5.56)	0 (0-5.30)	0 (0-5.90)
Primary care physician per 100,000 residents, median (IQR)	41.18 (27.3-58.8)	41.8 (27.6-60.9)	41.3 (26.9-59.6)	39.9 (25.0-58.9)	38.9 (24.2-57.7)	37.8 (22.9-57.3)
Persons 18-64 without health insurance, % , median (IQR)	15.0 (11.8-18.6)	15.0 (11.8-18.6)	19.6 (15.5-24.3)	21.4 (16.8-26.0)	21.2 (16.5-25.7)	14.2 (10.0-18.9)
Veterans, %, median (IQR)	13.8 (12.2-15.4)	13.8 (12.2-15.4)	11.5 (9.9-13.1)	11.1 (9.5-12.7)	10.0 (8.6-11.5)	8.9 (7.6-10.4)

© 2019 Steelesmith DL et al. *JAMA Network Open.*

Gun shops per 100,000 residents, median (IQR)	2.9 (0-6.2)	2.3 (0-5.5)	2.0 (0-5.1)	1.9 (0-5.1)	2.6 (0-5.9)	3.3 (0-7.0)
Drinking establishments per 100,000 residents, median (IQR)	20.6 (6.9-41.4)	18.2 (6.0-38.2)	16.8 (4.9-35.7)	16.4 (5.3-35.5)	14.2 (4.2-31.2)	11.7 (3.0-26.9)

Note: RUCC = Rural Urban Continuum Code

© 2019 Steelesmith DL et al. *JAMA Network Open.*

eTable 6. Longitudinal Random-Effects Models of Associations of Contextual Variables With Suicide Rates by Method From 1999 to 2016

	Firearm Suicides				All Other Suicides			
**Variables**	**IRR**	**95% CI**		**p-value**	**IRR**	**95% CI**		**p-value**
Median age[1]	1.002	0.999	1.005	.17	1.005	1.002	1.008	.001
Percent non-Hispanic white[1]	1.007	1.006	1.008	<.001	1.002	1.001	1.003	<.001
Percent male[1]	0.997	0.990	1.003	.28	1.016	1.010	1.023	<.001
Social fragmentation								
4th vs 1st quartile	1.032	0.999	1.066	.06	1.162	1.123	1.203	<.001
3rd vs 1st quartile	1.020	0.994	1.047	.14	1.130	1.098	1.163	<.001
2nd vs 1st quartile	1.017	0.995	1.040	.13	1.079	1.052	1.107	<.001
Social capital								
4th vs 1st quartile	0.827	0.796	0.859	<.001	1.051	1.010	1.094	.02
3rd vs 1st quartile	0.853	0.827	0.880	<.001	1.064	1.029	1.099	<.001
2nd vs 1st quartile	0.927	0.904	0.950	<.001	1.021	0.993	1.049	.14
Psychiatrist ratio, per 100,000 residents	0.994	0.993	0.996	<.001	1.002	1.000	1.004	.02
Primary care physician ratio, per 100,000 residents	1.000	1.000	1.001	.17	1.000	1.000	1.001	.33
Percent without health insurance	1.008	1.007	1.009	<.001	1.002	1.001	1.004	.002
Percent veterans	1.031	1.027	1.035	<.001	1.022	1.018	1.026	<.001
Drinking establishment ratio per 100,000 residents	1.000	0.999	1.000	.09	1.001	1.000	1.001	<.001
**Interaction Terms**								
Deprivation quartiles								
4th vs 1st quartile								
1999-2001, Large Metro	1.407	1.315	1.506	<.001	1.056	0.988	1.129	.11
1999-2001, Rural	1.594	1.422	1.786	<.001	1.188	1.044	1.353	.01
2014-2016, Large Metro	1.011	0.930	1.099	.80	0.919	0.845	0.999	.05
2014-2016, Rural	1.145	1.025	1.278	.02	1.034	0.912	1.173	.60
3rd vs 1st quartile								
1999-2001, Large Metro	1.242	1.184	1.304	<.001	1.015	0.966	1.066	.56
1999-2001, Rural	1.279	1.149	1.425	<.001	1.022	0.902	1.157	.73
2014-2016, Large Metro	1.028	0.971	1.089	.35	0.936	0.883	0.991	.02
2014-2016, Rural	1.059	0.954	1.175	.29	0.942	0.834	1.065	.34
2nd vs 1st quartile								
1999-2001, Large Metro	1.093	1.052	1.135	<.001	1.023	0.985	1.063	.24
1999-2001, Rural	1.127	1.015	1.252	.02	0.937	0.827	1.062	.32
2014-2016, Large Metro	1.045	1.004	1.087	.03	0.962	0.925	0.999	.05
2014-2016, Rural	1.078	0.973	1.194	.15	0.881	0.779	0.996	.04
Gun shops								
Large metro	1.033	1.025	1.042	<.001	0.992	0.984	1.001	.08
Small metro	1.014	1.009	1.019	<.001	1.006	1.000	1.011	.04

© 2019 Steelesmith DL et al. *JAMA Network Open.*

Micro	1.006	1.003	1.009	<.001	1.003	1.000	1.006	.08
Rural	1.003	1.000	1.006	.08	0.997	0.993	1.002	.20

[1] Control variables were global-mean centered, IRRs reflect 1 unit increase from average.

© 2019 Steelesmith DL et al. *JAMA Network Open.*

eMethods. Index Creation

Prior to examining how contextual factors impact county-level suicide rates, individual variables that measured similar constructs were reduced to indices through principal component analysis (PCA). PCA allows for the reduction of variables while retaining much of the overall variation in the data. Linear combinations of the original variables are used to create new variables, or principal components.[1,2] PCA was conducted separately for the socioeconomic, social fragmentation, and social capital variables described in eTable 3. After the PCA analysis, components were examined for meaning and interpretability which resulted in only a single component from each PCA being kept for the final analyses. The area deprivation index accounted for 38.6% of variance, the social fragmentation index accounted for 47.4% of variance, and the social capital index accounted for 28.5% of variance. While somewhat low for variance explained, these factors were modeled after previously used indices and only the first factor was retained since the factor loadings were as expected for interpretation of the indices. Factor scores for the components were computed for each county and time-period of the study with a mean of 0 and standard deviation of 1. All PCA analyses were done using SAS 9.4.3[3] Factor scores were then divided into quartiles based on the overall study, with roughly 25% of county/year categories falling into each quartile. The lowest quartile was used as a reference for all index variables.

Model selection

To examine associations between suicide and contextual variables, a series of longitudinal models using maximum likelihood estimation were used. For all models examined, the outcome was the count of suicides per county. The first step of the model building process examined unconditional means and unconditional growth models to measure the variation across counties and the variation across counties and time.[4] Next, contextual variables were examined in association with suicides. All contextual variables were included in the final model. Interactions between contextual variables and the four category rural urban continuum codes (RUCC) variable were examined to see how the associations of contextual variables with suicide varied across the rural/urban continuum. Interactions between contextual variables and time were also examined to explore how associations with suicide change over time. Only interactions that were significant at the .05 level were maintained in the final longitudinal model.[4] A single three-way interaction (time*RUCC*deprivation) was examined based on the significant two-way interactions, but this variable was not retained in the final model because it was not significant nor did it improve model fit.

© 2019 Steelesmith DL et al. *JAMA Network Open.*

eReferences

1. Abdi H, Williams LJ. Principal component analysis. *Wiley Interdiscip Rev Comput Stat*. 2010;2(4):433–459.

2. Jolliffe I. *Principal Component Analysis*. New York, NY: Springer Series in Statistics; 2002.

3. SAS Institute, Inc. *SAS Software*. Cary, NC: SAS, Inc; 2014.

4. Singer JD, Willett JB. *Applied Longitudinal Data Analysis: Modeling Change and Event Occurrence*. New York, NY: Oxford University Press; 2003.

© 2019 Steelesmith DL et al. *JAMA Network Open.*
